# Blood‐Based Biomarkers to Aid in Alzheimer’s Disease Prediction or Diagnosis: Analysis in a Multi‐Ethnic Cohort Study

**DOI:** 10.1002/alz.091795

**Published:** 2025-01-03

**Authors:** Aanya Bahl, Lawrence S. Honig, Min Suk Kang, Danurys Sanchez, Dolly Reyes‐Dumeyer, Jennifer J. Manly, Rafael A. Lantigua, Jeffrey L. Dage, Adam M. Brickman, Badri N. Vardarajan, Richard Mayeux, Yian Gu

**Affiliations:** ^1^ Department of Epidemiology, Mailman School of Public Health, New York, NY USA; ^2^ G. H. Sergievsky Center, Vagelos College of Physicians and Surgeons, New York, NY USA; ^3^ Columbia University Irving Medical Center, New York City, NY USA; ^4^ Columbia University Irving Medical Center, New York, NY USA; ^5^ Taub Institute for Research on Alzheimer’s Disease and the Aging Brain, Vagelos College of Physicians and Surgeons, Columbia University, New York, NY USA; ^6^ Department of Neurology, Vagelos College of Physicians and Surgeons, Columbia University, and the New York Presbyterian Hospital, New York, NY USA; ^7^ Columbia University, New York, NY USA; ^8^ The Gertrude H. Sergievsky Center, College of Physicians and Surgeons, Columbia University, New York, NY USA; ^9^ G. H. Sergievsky Center, Vagelos College of Physicians and Surgeons, Columbia University, New York, NY USA; ^10^ Columbia University Medical Center, New York, NY USA; ^11^ The Taub Institute for Research on Alzheimer’s Disease and the Aging Brain, Vagelos College of Physicians & Surgeons, Columbia University, New York, NY USA; ^12^ Taub Institute for Research on Alzheimer’s Disease and the Aging Brain, New York, NY USA; ^13^ Department of Neurology, Vagelos College of Physicians and Surgeons, Columbia University, New York Presbyterian Hospital, New York, NY USA; ^14^ Department of Neurology, Columbia University, New York, NY USA; ^15^ Taub Institute for Research on Alzheimer’s Disease and the Aging Brain, Columbia University, New York, NY USA; ^16^ Stark Neurosciences Research Institute, Indiana University School of Medicine, Indiana, IN USA; ^17^ Department of Neurology, The Taub Institute for Research on Alzheimer’s Disease and The Aging Brain, Columbia University Medical Center and The New York Presbyterian Hospital, New York, NY USA; ^18^ Departments of Neurology, Psychiatry, and Epidemiology, Gertrude H. Sergievsky Center, The Taub Institute for Research on Alzheimer’s Disease and the Aging Brain, Vagelos College of Physicians and Surgeons, Columbia University, New York, NY USA; ^19^ Mailman School of Public Health, Columbia University, New York, NY USA

## Abstract

**Background:**

Blood‐based biomarkers may aid in the diagnosis of Alzheimer’s Disease (AD), but their contribution may be confounded by the presence of multiple chronic conditions and have not been well‐tested in community populations. In the current study, we aimed to determine whether blood‐based biomarkers can aid in refining a multi‐ethnic, urban clinically diagnosed AD community‐based cohort.

**Method:**

We included 546 individuals in the Washington Heights, Hamilton Heights, and Inwood Columbia Aging Project (WHICAP) study in this cross‐sectional study. Six biomarkers, including phosphorylated‐tau‐181 (P‐tau181), total (T‐tau), amyloid‐beta 40 and 42 (Aβ40, Aβ42), Glial Fibrillary Acid Protein (GFAP), and Neurofilament Light Chain (NfL) were measured using Quanterix SIMOA HD‐X platforms. The association between the biomarkers and AD or cognitive impairment was tested using logistic regression, adjusted for age, sex, ethnic group, and years of education. Individuals were subsequently characterized as ‘biomarker positive’ or ‘biomarker negative’ based on combined GFAP and P‐tau181/Aβ42 cut scores.

**Result:**

The mean age of individuals was 79.3 years (6.56) and 379 (69.4%) were women, 133 (24.48%), were Non‐Hispanic Black, 153 (28.0%) Non‐Hispanic White, and 248 (45.4%) were Hispanic. A clinical diagnosis of AD was made in 129 (25.49%) individuals. Low Aβ42 (OR = 0.18, [95% CI: 0.04 ‐ 0.92]), low Aβ42/Aβ40 (OR = 0.49, [95% CI: 0.228 ‐ 0.872), and high P‐tau181/Ab42 (OR = 5.494, [95% CI: 1.523 – 20.416]) were associated with a clinical diagnosis of AD suggesting a role as predictive biomarkers. However, the best combination, GFAP and P‐tau181/Aβ42 cut scores, yielded a sensitivity of 41% and specificity of 70.5% for clinically diagnosed AD. The concordance was 54.5% and the discordance was present in both directions. Low education, cardiovascular and other comorbidities might contribute to the discrepancy between biomarker positivity and clinical diagnosis.

**Conclusion:**

While GFAP and P‐tau181/Aβ42 levels are associated with AD pathology and can aid in the diagnosis of AD, the presence of multiple chronic conditions may lead to either false positives or negatives. Large multi‐ethnic community cohort studies are needed to further examine the utility of these biomarkers in aiding in the clinical diagnosis of AD.

